# Isolation of axenic cyanobacterium and the promoting effect of associated bacterium on axenic cyanobacterium

**DOI:** 10.1186/s12896-020-00656-5

**Published:** 2020-11-30

**Authors:** Suqin Gao, Yun Kong, Jing Yu, Lihong Miao, Lipeng Ji, Lirong Song, Chi Zeng

**Affiliations:** 1 School of Biology and Pharmaceutical Engineering, Wuhan Polytechnic University, Wuhan, 430023 Hubei China; 2 College of Resources and Environment, Yangtze University, Wuhan, 430100 Hubei China; 3 Key Laboratory of Water Pollution Control and Environmental Safety of Zhejiang Province, Hangzhou, 310058 Zhejiang China; 4 Yixing Academy of Environmental Protection, Nanjing University, Yixing, 214200 Jiangsu China; 5 Yixing Urban Supervision & Inspection Administration of Product Quality, National Supervision & Inspection Center of Environmental Protection Equipment Quality (Jiangsu), Yixing, 214205 Jiangsu China; 6 Institute of Hydrobiology, Chinese Academy of Sciences, Wuhan, 430072 Hubei China

**Keywords:** *Microcystis aeruginosa*, Bacterial symbioses, Microbial ecology, Heterotrophic bacteria, Promoting effect

## Abstract

**Background:**

Harmful cyanobacterial blooms have attracted wide attention all over the world as they cause water quality deterioration and ecosystem health issues. *Microcystis aeruginosa* associated with a large number of bacteria is one of the most common and widespread bloom-forming cyanobacteria that secret toxins. These associated bacteria are considered to benefit from organic substrates released by the cyanobacterium. In order to avoid the influence of associated heterotrophic bacteria on the target cyanobacteria for physiological and molecular studies, it is urgent to obtain an axenic *M. aeruginosa* culture and further investigate the specific interaction between the heterotroph and the cyanobacterium.

**Results:**

A traditional and reliable method based on solid-liquid alternate cultivation was carried out to purify the xenic cyanobacterium *M. aeruginosa* FACHB-905. On the basis of 16S rDNA gene sequences, two associated bacteria named strain B905–1 and strain B905–2, were identified as *Pannonibacter* sp. and *Chryseobacterium* sp. with a 99 and 97% similarity value, respectively. The axenic *M. aeruginosa* FACHB-905A (*Microcystis* 905A) was not able to form colonies on BG_11_ agar medium without the addition of strain B905–1, while it grew well in BG_11_ liquid medium. Although the presence of B905–1 was not indispensable for the growth of *Microcystis* 905A, B905–1 had a positive effect on promoting the growth of *Microcystis* 905A.

**Conclusions:**

The associated bacteria were eliminated by solid-liquid alternate cultivation method and the axenic *Microcystis* 905A was successfully purified. The associated bacterium B905–1 has the potentiality to promote the growth of *Microcystis* 905A. Moreover, the purification technique for cyanobacteria described in this study is potentially applicable to a wider range of unicellular cyanobacteria.

## Background

The interactions between phototrophic phytoplankton and heterotrophic bacteria are considered to be an integral part of the algal/cyanobacterial life cycle. For example, diatoms and bacteria coexist in the ocean and coevolve in complex interactions that significantly modify each other’s behavior and ultimately impact biogeochemical cycles [1–3]. This interaction plays an important role in photosynthesis and is therefore crucial for the metabolism of phototrophic phytoplankton. The relations between phototrophic phytoplankton and heterotrophic bacteria are much better understood compared with that between zooplankton and bacteria, and it is generally recognized that there are three different types of phototrophic phytoplankton and heterotrophic bacteria interactions: (i) bacteria and phototrophic phytoplankton form a mutualistic relationship in which phytoplankton benefits from bacterial products such as nutrients, whereas bacteria profit from phytoplankton products such as extracellular polymeric substances [4]; (ii) bacteria and phototrophic phytoplankton form an antagonism relationship that the growth of phytoplankton is restricted or inhibited by bacteria through algal-bacterial/cyanobacterial-bacterial contact mechanism (direct interaction) or secretion of the extracellular antialgal/anticyanobacterial substances (indirect interaction) [5, 6] and (iii) bacteria and phototrophic phytoplankton form a commensal relationship that bacteria are loosely associated with phytoplankton and may promote the growth and photosynthesis without having any negative effect, while phytoplankton grows well without the associated bacteria [7, 8]. These scenarios may be dependent on the characteristics of phototrophic phytoplankton species, associated bacterial species and secreted substances of the associated heterotrophic bacteria [4].

Harmful cyanobacterial blooms (HCBs) in lakes, reservoirs and rivers have drawn great attention all over the world as microcystin-producing cyanobacteria cause animal and human health concerns [5, 6, 9]. *Microcystis aeruginosa*, a unicellular, photoautotrophic and gram-negative cyanobacterium that belongs to the genus *Microcystis*, division *Cyanophyta* is one of the most common and widespread bloom-forming cyanobacteria that secret toxins [5, 6, 10]. Previous studies show that the cyanobacterium is associated with a large number of bacteria, and these associated heterotrophic bacteria (heterotrophs) are considered to benefit from organic substrates released by the cyanobacterium [11–18]. In order to avoid the influence of heterotrophs for physiological and molecular studies, the purification of the axenic cyanobacterium (bacteria-free) is especially important as well as the understanding of its responses to the heterotrophs.

Various methods including UV irradiation, sonication, micropipette technique, phenol treatment, antibiotic treatment and lysozyme treatment have been used for cyanobacteria purification [19–25]. Previous study shows that treatment with antibiotics is a successful strategy to obtain axenic cyanobacteria cultures [1]. Additionally, solid medium is simple and useful for the growth and isolation of axenic *Microcystis* strains, in which way two axenic *Microcystis* strains are obtained [24, 26]. Although the direct and indirect inhibiting effects of bacteria on cyanobacteria have been intensively studied [3, 5, 6, 12, 13, 27] and the associated bacteria are potentially regarded to regulate cyanobacterium growth via extracellular amino acid monomers or other substances [5, 6], the growth-promoting effects of heterotrophs on cyanobacterium have not received much attention. Apart from cyanobacterium purification, the growth-promoting effect of the heterotrophs on cyanobacterium is a significant aspect for understanding the interactions between heterotrophs and cyanobacteria. Therefore, the aim of the present study is to obtain an axenic *M. aeruginosa* culture and investigate the specific interaction between the heterotrophs and the cyanobacterium.

## Results

### Isolation and purification of the axenic culture

**Fig. 1:**
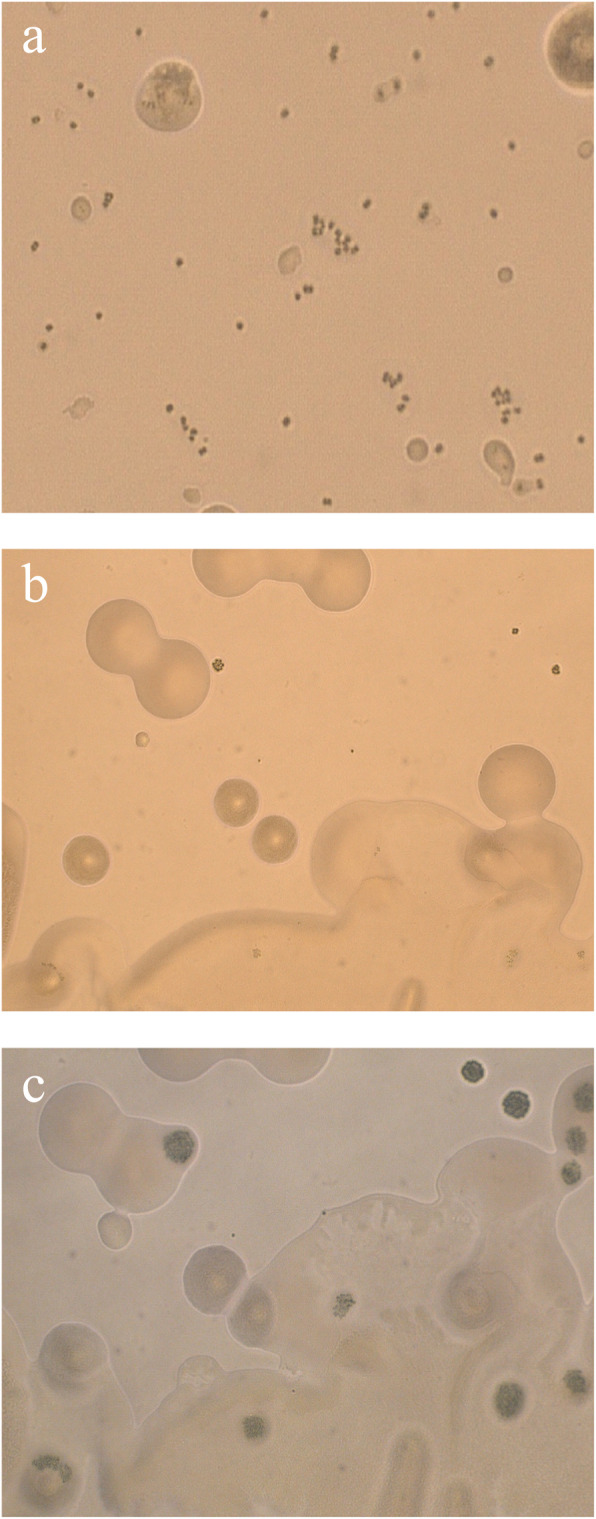
The growth of cyanobacterial and heterotrophic colonies (× 100). (**a**, **b** and **c** was the colonial morphology cultured for 1, 8 and 15 d, respectively)

*M. aeruginosa* 905 and 907 samples were curated by the Freshwater Algae Culture Collection of Institute of Hydrobiology (FACHB) as xenic consortia comprised of one *M. aeruginosa* strain and its associated heterotrophic bacteria. The colony forming process of cyanobacterium and heterotrophs on solid plates (BG_11_ agar medium) was observed by inverted phase contrast microscope, and the results were shown in Fig. 1. It is obvious that the heterotrophs colonies were much bigger than the cyanobacterium colonies, indicating the heterotrophs were grew much better compared with cyanobacterium. The cyanobacterium colony was formed when cultured for 15 d, although it was small; moreover, the cyanobacterial colonies were only found in 3 plates among the 20 replicate plates even after incubating for 20 d. Then the isolated cyanobacterial colonies were transferred into 6 test tubes with a Pasteur pipette under the microscope and incubated for 3 d. The result showed that 5 tubes become green, indicating the cyanobacterium grew well. With several cycles of purification, the axenic *M. aeruginosa* FACHB-905A (*Microcystis* 905A) was obtained. Possible contamination such as heterotrophs was subsequently examined before and after the incubations, and the results revealed that there was no contamination. Then a molecular identification was carried out for the purified axenic cyanobacterium named as *Microcystis* 905A. The results indicated that *Microcystis* 905A presented the highest sequence similarity (99% of identity) with *M. aeruginosa* NIES-843, *M. aeruginosa* PCC 7820 and *M. aeruginosa* PCC 7806.

### Identification of associated bacteria

**Fig. 2:**
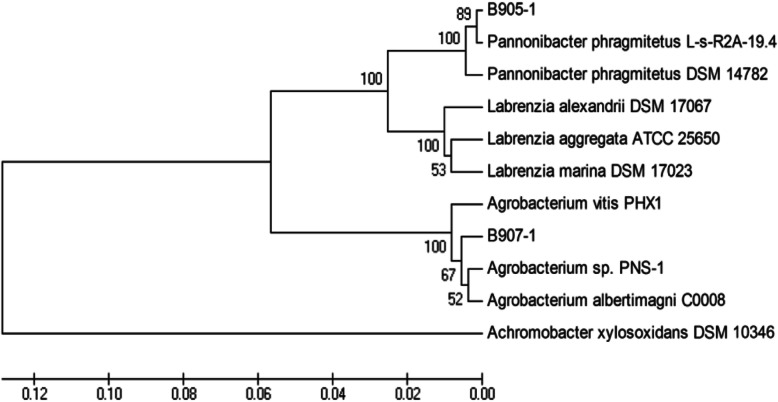
The phylogenetic tree of heterotrophs

Two gram-negative bacteria, named B905–1 and B905–2, were isolated from the xenic *M. aeruginosa* FACHB-905 (*Microcystis* 905). To identify the bacteria, phylogenetic analyses were performed using the 16S rDNA sequences. A total of 1367 bp of each of the two isolated strains was determined, and the 16S rDNA gene sequences obtained were subjected to GenBank BLAST search analyses [28]. Strain B905–1 was most closely related to *Pannonibacter phragmitetus* L-s-R2A-19.4 with a 99% similarity value, and strain B905–2 was most closely related to *Chryseobacterium* sp. with a 97% similarity value. With the same method, the xenic *M. aeruginosa* FACHB-907 (*Microcystis* 907) was diluted and then plated on the BG_11_ solid medium. After culturing under the culture conditions (Section “Culture of cyanobacteria and heterotrophs”) for 15 ~ 20 d, the single cyanobacterial colonies were transferred into test tubes with a Pasteur pipette under the microscope and incubated for 3 d. The results showed that 5 tubes became green, indicating the cyanobacterium grew well. With several cycles of purification, the axenic *Microcystis* 907A and another heterotroph B907–1 were also successfully isolated, and B907 was identified as *Agrobacterium* sp., which was most closely related to *Agrobacterium* sp. PNS-1 and *Agrobacterium albertimagni* C0008 with a 98% similarity value. The sequences of B905–1 and B907–1 were imported into the DNAMAN software V6 and aligned [29]. Phylogenetic tree was then constructed (Fig. 2) and it was further confirmed that strain B905–1 and B907–1 were closely related to *Pannonibacter* sp. and *Agrobacterium* sp., respectively.

### Effect of associated bacteria on *Microcystis* 905A

**Fig. 3:**
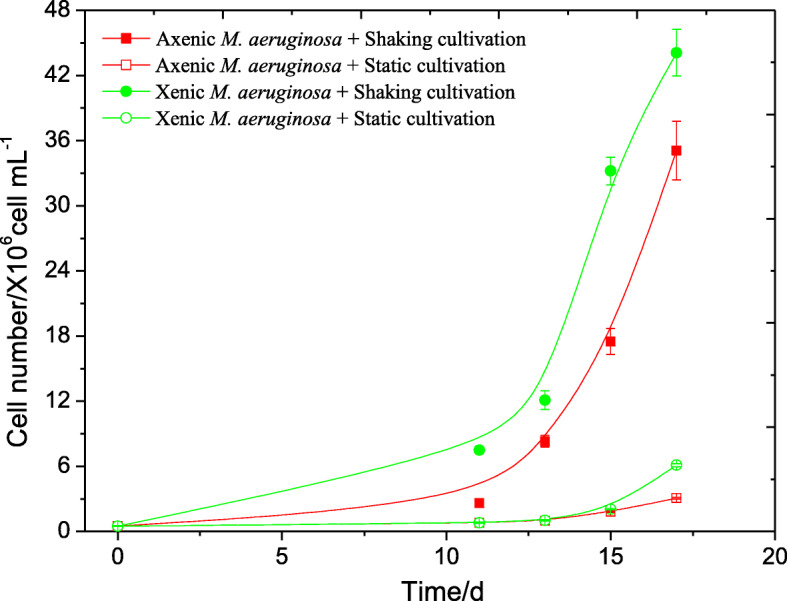
The growth curves of axenic *Microcystis* 905A and xenic *Microcystis* 905

The growth rates of xenic culture (*Microcystis* 905) and axenic culture (*Microcystis* 905A) were measured under both the static cultivation (without the shaking speed) and the shaking cultivation conditions (with the shaking speed of 150 rpm). Figure 3 indicated that the generation time of axenic culture was 42.3 h (shaking cultivation) and 60.9 h (static cultivation), while the generation time of xenic culture was 33.6 h under the shaking cultivation and 45.3 h under the static cultivation, respectively. In addition, the generation time of xenic culture was much shorter than that of the axenic culture under the same cultivation condition, which demonstrated the photosynthetic efficiency of *Microcystis* 905 was much better. At the same time, the growth rates of both the xenic culture and axenic culture under the shaking cultivation condition were much faster than that under the static cultivation condition. These results pointed to the role of the heterotrophs in promoting the growth of *Microcystis* 905A.

### Effect of heterotroph-cyanobacterium ratio on *Microcystis* 905A

**Fig. 4:**
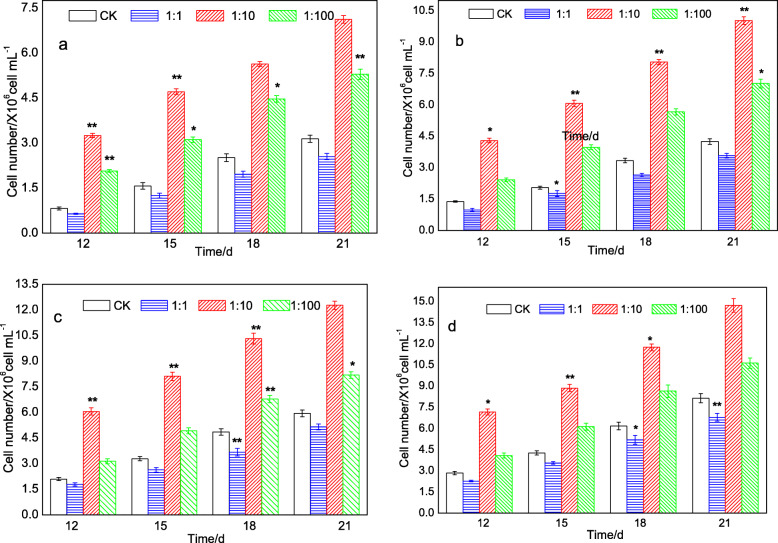
Effects of heterotroph-cyanobacterium ratio on axenic *Microcystis* 905A (**a**, **b**, **c** and **d** showed the initial cyanobacterial cell number of 3.0 × 10^2^, 3.0 × 10^3^, 3.0 × 10^4^ and 3.0 × 10^5^ cell mL^− 1^, respectively). * and ** represented a statistically significant difference of *p* < 0.05 and *p* < 0.01 when compared to the control

To further study the effect of heterotroph B905–1 on the growth of axenic *Microcystis* 905A, a series of experiments that different initial cyanobacterial cell concentrations with the heterotroph-cyanobacterium ratio of 1:1, 1:10 and 1:100 were undertaken in BG_11_ liquid medium (Fig. 4). Compared with the control group (CK), the cyanobacterial cell numbers of 1:1 treatment group was slightly suppressed during the 21 d, while the 1:10 and 1:100 treatment groups showed a remarkable increase, and they were increased with the extension of culture time. In addition, the highest cyanobacterial cell number for the treatment group of 1:10 and 1:100 was (14.72 ± 0.48) × 10^6^ cell mL^− 1^ and (10.63 ± 0.37) × 10^6^ cell mL^− 1^, respectively (Fig. 4d), and both of them were obtained at the 21st day. Obviously, the cyanobacterial cell numbers for the 1:10 treatment group were much higher than that for the 1:100 treatment group under the same conditions, and the reason might due to the higher concentration of the strain B905–1 that added at the beginning of the experiment. These results indicated the addition of heterotroph B905–1 had a positive promoting effect on the growth of *Microcystis* 905A.

**Fig. 5:**
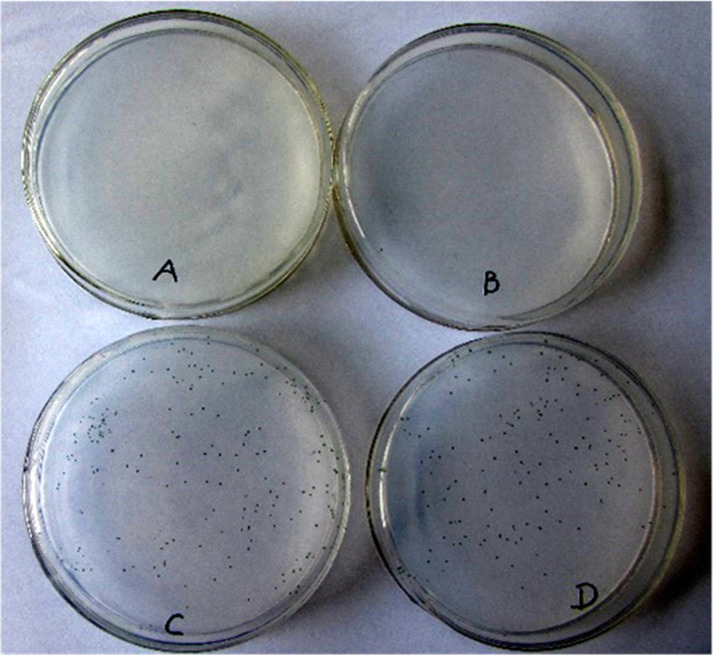
Effects of strain B905–1 on axenic *Microcystis* 905A cultured by plate. (**a** was the control without the addition of strain B905–1; **b**, **c** and **d** was the treatment with the addition of strain B905–1 at an initial cell number of 1.0 × 10^4^, 1.0 × 10^3^ and 1.0 × 10^2^ cell mL^− 1^, respectively)

The growth of axenic *Microcystis* 905A on BG_11_ agar medium with and without the addition of strain B905–1 was also investigated. For the treatments that with the addition of strain B905–1, the cyanobacterial colony of axenic *Microcystis* 905A became green after incubating for 20 days; while for the treatments that without the addition of strain B905–1, there was no cyanobacterial colony on BG_11_ agar medium. Moreover, the effects of different heterotroph-cyanobacterium ratio (1:1, 1:10 and 1:100) on the growth of cyanobacterium on BG_11_ agar medium were studied (Fig. 5). Interestingly, the *Microcystis* 905A was unable to grow in the treatment of 1:1 (Fig. 5b), but it grew well in both treatments of 1:10 and 1:100 (Fig. 5c and d). The results indicated high ratio of heterotroph-cyanobacterium (1:1) was not good for the growth of *Microcystis* 905A, which meant when the initial concentrations of B905–1 and axenic *M. aeruginosa* were the same, the growth of *M. aeruginosa* on both BG_11_ liquid medium and BG_11_ agar medium were inhibited. Previous study showed BG_11_ could become carbon- or phosphate-limited in dense cultures for some cyanobacteria [30]. The growth of *Microcystis* 905A was best in the 1:10 condition indicated the C-P ratios influenced by B905–1 were best balanced, where the heterotroph produced an enhancing amount of CO_2_, but didn’t consume too much phosphate in competition with *Microcystis* 905A.

**Fig. 6:**
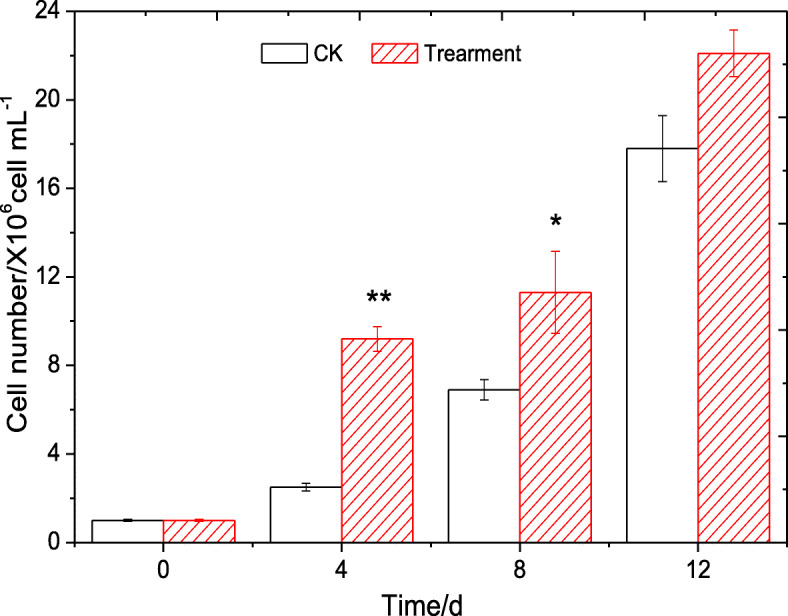
Effect of cell-free filtrate of strain B905–1 on axenic *Microcystis* 905A. * and ** represented a statistically significant difference of *p* < 0.05 and *p* < 0.01 as compared with the control

In order to prove that the promoting effect was associated with the extracellular substances of strain B905–1, the effect of the cell-free filtrate of strain B905–1 on the growth of *Microcystis* 905A was carried out (Fig. 6). Results showed that the cyanobacterium cell number of the treatment with the addition of the cell-free filtrate was 9.23 ± 0.56, 11.31 ± 1.85 and 22.14 ± 1.06 cell mL^− 1^ after incubating for 4 d, 8 d and 12 d, respectively, and it was obviously higher than that with no cell-free filtrate. The axenic cyanobacterium grew much better with the addition of the cell-free filtrate again demonstrating strain B905–1 had the promoting effect on the growth of *Microcystis* 905A; moreover, the released substances of strain B905–1 with the promoting effect were apparently existed in the cell-free filtrate.

## Discussion

HCBs occur around the world and are responsible for most aquatic environment pollution [9, 10]. Researches of HCBs have been concentrated on the physical, chemical and bio-ecological methods for the control of cyanobacteria and the removal of nitrogen and phosphorous [5, 9]. Little is known about the microbial community of cyanobacteria with heterotrophs and the interactions between them [2, 3]. Previous studies demonstrated that the oxic cyanobacterial layer of eutrophic water was mainly composed by cyanobacteria and aerobic heterotrophic microorganisms, and the relationships between them were complicated [31, 32]. Therefore, it is necessary to obtain the axenic *M. aeruginosa* from the complex microbial community and further research the interactions between cyanobacteria and heterotrophs.

Traditionally, cyanobacterial purification methods including antibiotic treatment and lysozyme treatment had been applied for eliminating heterotrophs from cyanobacteria and algae [20, 21, 25, 33], and the purification effects were depended on the concentrations and types of antibiotic or lysozyme [22–25]. With a series of antibiotic and lysozyme procedures, the axenic cyanobacteria such as *Anabaena flos-aquae*, *Aphanothece nidulans*, *Arthrospira platensis* and *Arthrospira* spp. were obtained [22, 23]. While the sensitivities of xenic cyanobacterium *Microcystis* 905 to five antibiotics employed in the present study are quite different, in particular, four of the tested antibiotics have the inhibition effects on cyanobacterium growth. Furthermore, the lysozyme can inhibit both the cyanobacterium and heterotroph simultaneously (Supporting Information of Table S1 and Fig. S1), it is quite difficult to eliminate the heterotrophs from xenic *Microcystis* 905 culture by antibiotics treatment or lysozyme treatment methods. Researches indicate the bloom forming cyanobacteria in freshwater or seawater are more often occurred in nutrient-rich environments, and the cyanobacteria are surrounded by diverse communities of heterotrophic bacteria [31, 32, 34]. The difficulty in obtaining the axenic *Microcystis* 905 is probably due to the lack knowledge of heterotrophs in xenic culture.

Heterotrophs can colonize within the enclosed region or directly adhere to the surface of a cyanobacterium colony [34]. By transferring and culturing xenic culture of *Arthrospira platensis* in fresh sterile medium, the axenic *A. platensis* is obtained by the technique of single-trichome manipulation performed with a microtrowel [35]. Considering the xenic *Microcystis* 905 can easily form single cyanobacterial colony on BG_11_ agar plate and the growth rates of *Microcystis* and heterotrophs are significantly different, heterotrophs are removed by solid-liquid alternate cultivation method and micropipette technique, which by picking and transferring the single cyanobacterial colony to BG_11_ liquid medium under the microscope. This method not only guarantees the minimum initial growth density of cyanobacterial cells, but also ensures the purity of cyanobacterial cells, thus results in the successful separation of the axenic *Microcystis* 905A. It is also successfully applied to purify other strain such as axenic *Microcystis* 907A. In spite of the traditional standard plate method based on solid-liquid alternate cultivation for obtaining axenic culture is time-consuming, the protocol that we have developed for purifying axenic *Microcystis* 905A culture maybe suitable for separating axenic strains from a commensal, and potentially syntrophic, symbiosis. These results indicate that this technique is at least applicable to unicellular cyanobacteria.

Molecular biological techniques such as denaturing gradient gel electrophoresis (DGGE) and fluorescence in situ hybridization have been used to investigate the purity of cyanobacterial culture [17, 30]. DGGE results suggest that a number of bacteria including α-*proteobacteria*, β-*proteobacteria*, γ-*proteobacteria*, *Bacteroidetes* and *Actinobacteria* have been detected in the cyanobacterial cultures, and the *Sphingomonadales* are the prevalent group among the *Microcystis*-associated bacteria [17]; in another study, the heterotrophs, for instance, *Aeromicrobium alkaliterrae*, *Halomonas desiderata* and *Staphylococcus saprophyticus* are also identified from the *Arthrospira platensis* culture [25]. The heterotrophic bacteria, such as α-*proteobacteria* and bacteria from the *Bacteroidetes*-group, are reported to associate with Diatoms in nature as well as in stock cultures [1]. We observe that the heterotrophs strain B905–1 and B905–2 are closely related to *Pannonibacter* sp. and *Chryseobacterium* sp., respectively. Besides the identification of heterotrophs, it seems that more attention should be paid to the interactions between heterotrophs and the cyanobacterium *M. aeruginosa*. It is suggested that the interaction between heterotrophs and cyanobacterium is symbiosis or parasitic [3, 36], and the heterotrophs are difficult to isolate from cyanobacterium during the formation of cyanobacterial or algal colony [1, 37].

Heterotrophs can enhance or suppress the growth of cyanobacteria, or even kill them [31, 34]. To better understand the general interaction between heterotroph and cyanobacterium, the effect of the strain B905–1 on the cyanobacterium *M. aeruginosa* FACHB-905A is studied. It is showed that the growth rate of the xenic *Microcystis* 905 is much faster than that of the axenic xenic *Microcystis* 905A under both static cultivation and shaking cultivation conditions. The results indicate that the heterotroph B905–1 has a promoting effect on the growth of axenic *Microcystis* 905A. In consideration of the initial cell number of *Microcystis* 905 is (2.2 ± 0.2) × 10^6^ cell mL^− 1^ and heterotroph B905–1 is (0.64 ± 0.07) × 10^6^ cell mL^− 1^, it is not surprising that the growth-promoting effect of the 1:10 treatment is much better than the 1:100 treatment. Interestingly, the *Microcystis* 905A is unable to form colonies in the 1:1 treatment group on BG_11_ agar medium. Although *M. aeruginosa* is a kind of photosynthetic bacterium (or autotrophic bacteria) and it grows well under the light with inorganic nutrients, which are supplied by BG_11_ liquid medium, it is not surprising that axenic *Microcystis* 905A could not divide at the heterotroph-cyanobacterium ratio of 1:1, as the heterotroph B905–1 can effectively compete nutrients with axenic *Microcystis* 905A.

The growth-promoting effect of heterotrophs on algae has recently been observed in other studies, for example, the growth of toxic dinoflagellate *Alexandrium fundyense* is promoted substantially by *Alteromonas* sp. [8], and the attached bacteria provide co-existing for diatom *Thalassiosira weissflogii* to form transparent exopolymer particles [4]. Interpretation of such phenomenon might be explained by the symbiotic interaction that the bacteria deliver vitamins for algae [38], or the addition of bacteria changes the available nutrient concentration such as extracellular organic carbon or dissolved organic matter [2, 4, 14, 17, 31]. In a previous study, the growth rate and metabolic products of *Shewanella putrfaciens*, *Brochothrix thermosphacta* and *Pseudomonas* sp. show a remarkable increase no matter cultured individually or in all possible combinations compared to the control cultures [39]. Difference from the above-mentioned microorganisms, axenic diatoms are unable to form biofilm when purified from bacteria [4]. Although the axenic *Microcystis* 905A grows well under the liquid culture condition, it could not form cyanobacterial colonies on the BG_11_ agar plate without the addition of strain B905–1, indicating the presence of heterotroph B905–1 is indispensable for the growth of axenic *Microcystis* 905A on BG_11_ agar plate. The different growth phenomenon of *Microcystis* 905A in solid and liquid BG_11_ medium is mainly attributed to the phosphate. It is reported that reactive oxygen species (ROS) were produced when phosphate was autoclaved together with agar, and total colony counts of *Gemmatimonas aurantiaca* in liquid medium (without agar) were remarkably higher than those grown on solid medium (with agar) [40]. In the same way, there may be some ROS produced in BG_11_ solid medium and the ROS is likely a contributing factor to the growth inhibition of *Microcystis* 905A. It is speculated that the heterotrophic bacterium B905–1 closely associated with cyanobacterium likely consume nutrients that released by *Microcystis* 905, and may also produce vitamins and other beneficial metabolites useful for cyanobacterial growth [32, 34]. Nevertheless, the presence of strain B905–1 for the cyanobacterial colony formation mechanism needs to be further studied.

Previous study also indicates that the enhancement growth of axenic *Microcoleus chthonoplastes* PCC 7420 is upon the addition of a filtrate obtained from the closely related xenic culture of *Microcoleus* sp. M2C3, and the stimulated effect could be due to the release of certain growth factors and vitamins by associated aerobic heterotrophic microorganisms [31]. Most of the strains are able to secrete active substance to inhibit or enhance the growth of cyanobacteria [41]. Possible mechanisms may include various types of interactions from nutrient cycling to the production of growth-inhibiting and cell-lysing compounds [42]. Our results demonstrate that strain B905–1 has the potential to promote *Microcystis* 905A growth, whereas *Microcystis* 905A provides organic matter for associated bacterial proliferation. In a comparable study it is pointed out that bacteria have the potential to control diatom growth, and their interactions are regulated by multiple signals involving common biomolecules such as proteins, polysaccharides and respective monomers [14]. In accordance with previous observations, we also find the associated bacterium has promoting effect on the growth of cyanobacterium *M. aeruginosa*. Increasing knowledge on molecular mechanisms of microbial interactions are crucial to better understand or predict nutrient and organic matter cycling in aquatic environment, and also to better understand the role of such associated bacterium for the formation mechanism of HCBs and eutrophication control.

Up to now, most studies on the interaction between heterotrophs and cyanobacteria are performed in pure cultures [32, 34, 41], and the growth of the axenic cyanobacteria is almost promoted by the heterotrophs [8, 32, 34]. However, the interaction can be profoundly different in nature, as most microbes are not axenic but grow together in communities. The complex communities or microbial networks often result in surprisingly coordinated multicellular behaviour, e.g. dinoflagellates can feed on associated bacteria and heterotrophs also attack and lysis the cyanobacteria [31]. Furthermore, the heterotrophs are considered as playing a significant role in carbon cycling and cyanobacterial photosynthesis [31]. All these studies suggest that the relationship between heterotrophs and cyanobacteria in nature is complex and manifold, further analysis is needed to have a full understanding of the microbial communities surrounding cyanobacteria.

## Conclusions

Our results showed that heterotrophs were eliminated by solid-liquid alternate cultivation method and the axenic *Microcystis* 905A was successful purified by means of picking and transferring the single cyanobacterial colony to BG_11_ liquid medium under the microscope; moreover, two heterotrophs, strain B905–1 and strain B905–2, were identified as *Pannonibacter* sp. and *Chryseobacterium* sp. with a 99 and 97% similarity value in the basis of 16S rDNA gene sequences. Further, strain B905–1 had the potentiality to promote the growth of *Microcystis* 905A. The purification technique for cyanobacteria described in this study is potentially applicable to a wider range of unicellular cyanobacteria.

## Methods

### Culture of cyanobacteria and heterotrophs

Xenic *Microcystis* 905 and *Microcystis* 907 used in this study were purchased from the FACHB, Chinese Academy of Sciences (Wuhan, China). Sterilized BG_11_ liquid medium or BG_11_ agar medium (with the agar concentration of 1.5%) was used as the main culture medium for both axenic and xenic *M. aeruginosa* [5, 6, 43]. Before being used as inoculants, cyanobacteria were cultured with 200 mL BG_11_ liquid medium in 500 mL Erlenmeyer flasks for 7 days to reach the log phase, and the culture conditions were as follows: 2000 lx white light, light: dark = 14 h: 10 h; 25 ± 1 °C [5, 6]. Axenic *Microcystis* 905A was obtained by treating with micro-picking from *Microcystis* 905 culture.

Bacterial strains B905–1 and B905–2 were isolated from the culture solution of the cyanobacterium *Microcystis* 905. These two bacteria were routinely grown in TY liquid medium [44] at 28 ± 1 °C under aerobic conditions (with the shaking speed of 150 rpm). The cell-free filtrate of strain B905–1 was obtained by centrifuging the fermentation broth at 10,000×g for 10 min and then filtered through with the 0.22 μm cellulose acetate membrane [5]. Stock cultures were kept at 4 °C, and working cultures were obtained from stock cultures through two transfers in appropriate TY liquid medium.

### Isolation and purification of axenic culture

**Fig. 7:**
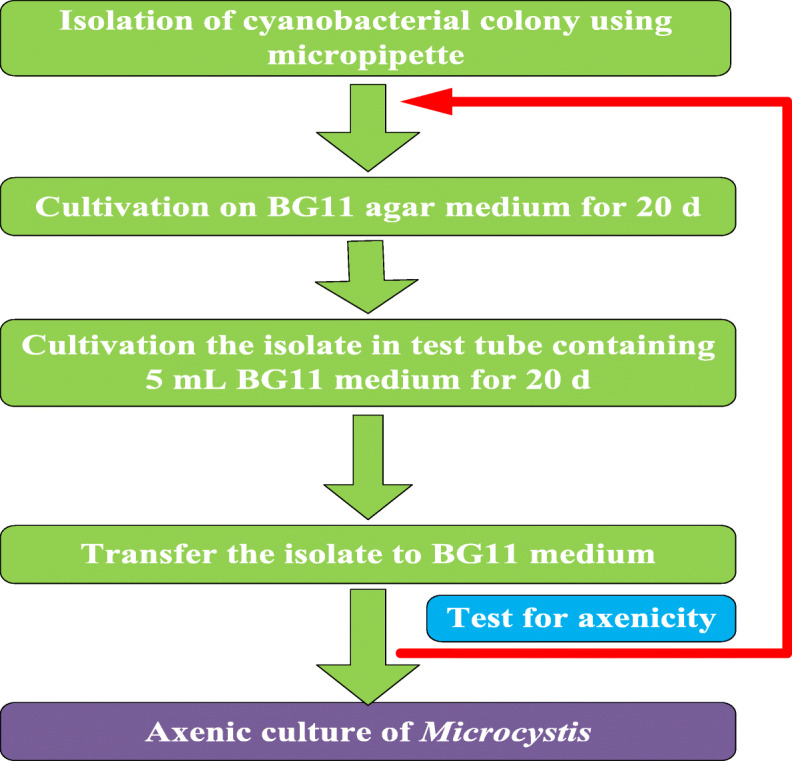
Purification procedure for axenic culture of *Microcystis*

For the isolation and purification of axenic cultures, cyanobacterial cells were treated by the solid-liquid alternate cultivation method. The xenic cyanobacterium was diluted to different multiple from 10^− 1^ to 10^− 8^, and the different multiple were inoculated onto sterile Petri dishes containing BG_11_ agar medium, respectively [45]. After incubating for 15 to 20 d under the culture conditions above, a single cyanobacterial colony was picked by a Pasteur pipette with the aid of a microscope, and then transferred into a test tube that containing 5 mL BG_11_ liquid medium. The purification result was checked as the test tube becoming green, and the testing method was as follows: 0.1 mL cyanobacterial culture from the test tube was spread on the Luria-Bertani (LB) agar plate [44, 46] and incubated at room temperature for 3 d or more to examine the existence of heterotrophs, the absence of heterotrophs indicated this cyanobacterial culture was axenic. After the purification, the axenic cyanobacterial colony was picked up by a Pasteur pipette, then transferred to Erlenmeyer flasks with BG_11_ liquid medium, and incubated at 25 ± 1 °C in a 14 L/10D light-dark cycle. The purification procedure for axenic *Microcystis* was illustrated in Fig. 7.

### Cyanobacterial inhibition bioassay

The growth curve of axenic *Microcystis* 905A and xenic *Microcystis* 905 were carried out at an initial cyanobacterial cell number of 1.0 × 10^6^ cell mL^− 1^. Effects of heterotroph-cyanobacterium ratio on the growth of four kinds of different initial axenic cyanobacterial cell concentrations in BG_11_ liquid medium were performed as follows: the axenic *Microcystis* 905A was firstly added in 250 mL sterilized Erlenmeyer flasks containing 100 mL BG_11_ liquid medium to keep the cyanobacterial cell number of 3.0 × 10^2^, 3.0 × 10^3^, 3.0 × 10^4^ and 3.0 × 10^5^ cell mL^− 1^, respectively, and then strain B905–1 (initial cell number was 2.73 × 10^7^ cell mL^− 1^) was added according to heterotroph-cyanobacterium ratio of 1:1, 1:10 and 1:100, the controls (CK) were without the addition of strain B905–1. For the effects of heterotroph-cyanobacterium ratio on the growth of axenic *Microcystis* 905A on BG_11_ agar medium, the heterotroph (B905–1) and cyanobacterium (axenic *M. aeruginosa*) were mixed well in BG_11_ liquid medium, and the final heterotroph-cyanobacterium ratios of 1:1, 1:10 and 1:100 were performed by adding different amounts of bacterium into the 100 mL axenic *M. aeruginosa* culture with the initial cyanobacterial cell number of 1.0 × 10^4^ cell mL^− 1^. The mixed suspensions were diluted to different multiples and then plated on the BG_11_ agar medium, each dilution gradient was repeated for three times.

The effect of cell-free filtrate of strain B905–1 on axenic *Microcystis* 905A was carried out by adding the cell-free filtrate (2%, v/v) into a 100 mL sterilized Erlenmeyer flask which containing initial axenic cyanobacterial cell number of 1.0 × 10^6^ cell mL^− 1^. The cell-free filtrate was obtained by filtrating with the 0.22 μm cellulose acetate membrane. The negative control was made by adding the same amount of TY liquid medium into 100 mL cyanobacterial culture or BG_11_ agar plate.

All the experiments were performed under aseptic conditions, the controls (CK) and the treatments were replicated three times, and the arithmetical means (± SD) were used as the final results.

### DNA extraction, sequencing and phylogenetic analysis

The isolated bacterial strains were identified based on 16S rRNA gene sequence analysis. Heterotrophs were prepared by incubating the seed culture at 37 °C with a shaking speed of 180 rpm for 20 h in sterilized LB liquid medium. The heterotroph cells were collected by centrifugation at 4000 rpm for 10 min (at 4 °C). DNA was extracted from the bacterial sample using the 3S DNA Isolation Kit V2.2 (Biocolor BioScience & Technology Co., Shanghai, China). Fragments of the 16S rDNA were amplified by PCR using the primers 27F (5′-GAGTTTGATCCTGGCTCAG-3′) and 1492R (5′-ACGGCTACCTTGTTACGACTT-3′), and the amplified fragments were sequenced by AuGCT Biotech Co., Ltd. (Beijing, China) [17]. The BLAST procedure was used to search for sequence similarity in GenBank [28].

### Analysis methods

Bacteria cell density is determined by colony counting method. Samples are cultured on TY agar medium at 28 ± 1 °C for 48 h, and the colonies are counted. The cyanobacterium cell number is determined by hemocytometer using light microscopy (NIKON-YS100). The cell density or cell number of each sample is counted in triplicate, and standard error of the mean is calculated for all data. Statistical analysis is performed using Version 17.0 of SPSS for Windows (SPSS, Chicago, IL, USA) [6].

The generation time (G) of the cyanobacterium is calculated according to eq. (1):

1 $$ \mathrm{G}=\left({\mathrm{t}}_2-{\mathrm{t}}_1\right)/\left[3.322\left({\mathrm{lgX}}_2-{\mathrm{lgX}}_1\right)\right] $$

where X_1_ and X_2_ are the cyanobacterium cell number at time t_1_ and t_2_, respectively.

The inhibition efficiency is calculated according to eq. (2):

2 $$ \mathrm{Inhibition}\ \mathrm{efficiency}=\left(1-{\mathrm{C}}_{\mathrm{t}}/{\mathrm{C}}_0\right)\times 100\% $$

where C_0_ and C_t_ are the cyanobacterium cell number of the control and test group at time t, respectively [5, 6].

## Supplementary Information

**Additional file 1: Table S1.** Effects of antibiotics on heterotrophs and cyanobacterium. **Figure S1.** Effects of lysozyme on heterotrophs and *Microcystis* 905.

## Data Availability

The data are presented within the manuscript and the cyanobacteria such as *M. aeruginosa* FACHB-905 and FACHB-907 used in this study could be purchased from the Freshwater Algae Culture Collection of Institute of Hydrobiology (FACHB), Chinese Academy of Sciences (Wuhan, China).

## Abbreviations

HCBs: Harmful cyanobacterial blooms
*Microcystis* 905A: The axenic *M. aeruginosa* FACHB-905A
*Microcystis* 907A: The axenic *M. aeruginosa* FACHB-907A
*Microcystis* 905: The xenic *M. aeruginosa* FACHB-905
*Microcystis* 907: The xenic *M. aeruginosa* FACHB-907
FACHB: Freshwater Algae Culture Collection of Institute of Hydrobiology
DGGE: Denaturing gradient gel electrophoresis
