# Supplementary material for: Isolation of axenic cyanobacterium and the promoting effect of associated bacterium on axenic cyanobacterium
Source: BMC Biotechnol. 2020 Nov 30;20:61. doi: 10.1186/s12896-020-00656-5 (PMC7708224; doi:10.1186/s12896-020-00656-5)
Supplement: Supplementary file 1 — Additional file 1: Table S1. Effects of antibiotics on heterotrophs and cyanobacterium. Figure S1. Effects of lysozyme on heterotrophs and Microcystis 905. [file 12896_2020_656_MOESM1_ESM.docx]

Isolation of axenic [cyanobacteri](app:ds:cyanobacteria)um and the promoting effect of associated bacterium on axenic [cyanobacteri](app:ds:cyanobacteria)um

Suqin Gao^1*^, Yun Kong^2,3,4,6*^, Jing Yu^1^, Lihong Miao^1**^, Lipeng Ji^2^, Lirong Song^5^, Chi Zeng^1^

1. School of Biology and Pharmaceutical Engineering, Wuhan Polytechnic University, Hubei Wuhan 430023, China

2. College of Resources and Environment, Yangtze University, Hubei Wuhan 430100, China

3. Key Laboratory of Water Pollution Control and Environmental Safety of Zhejiang Province, Zhejiang Hangzhou 310058, China

4. Yixing Academy of Environmental Protection, Nanjing University, Jiangsu Yixing 214200, China

5. Institute of Hydrobiology, Chinese Academy of Sciences, Hubei Wuhan 430072, China

6. Yixing Urban Supervision & Inspection Administration of Product Quality, National Supervision & Inspection Center of Environmental Protection Equipment Quality (Jiangsu), Jiangsu Yixing 214205, China

*These authors contributed equally to this manuscript.

**Corresponding author. Tel.: +86-27-83956793; Fax: +86-27-83955611

E-mail address: [ky020241@hotmail.com](mailto:ky020241@hotmail.com); [lhmiao2002@aliyun.com](mailto:lhmiao2002@aliyun.com);

Effects of antibiotics and lysozyme on [the xenic cyanobacteri](app:ds:cyanobacteria)um

The antibiotics (including tetracycline, cephalosporin, kanamycin, penicillin and streptomycin) and lysozyme were purchased from Wuhan Dingguo biological technology Co., LTD. The sensitivities of heterotroph to tetracycline, cephalosporins, kanamycin, penicillins and streptomycin were evaluated by the [filtering paper method](app:ds:filtering%20paper%20method) with the final concentration of 100 µg mL^-1^ for each treatment. The sensitivities of cyanobacterium to five antibiotics were carried out by adding antibiotic with the final concentration of 100 µgmL^-1^ in 250 mL sterilized Erlenmeyer flasks containing 100 mL xenic *Microcystis* 905 culture (the initial cyanobacterial cell number was 1.0 × 10^6^ cell mL^-1^), and the controls (CK) were added without any antibiotic. Effects of lysozyme on the cyanobacterium and heterotrophs were performed by adding lysozyme (the lysozyme was dissolved with sterile distilled water and then ﬁltered through with a 0.22 μm membrane) at ﬁnal concentrations of 0, 1.0, 2.0, 5.0, and 10.0 mg mL^-1^ in 250 mL sterilized Erlenmeyer flasks containing 100 mL xenic *Microcystis* 905 culture (the initial [cyanobacteri](app:ds:cyanobacteria)al cell number was 1.0 × 10^6^ cell mL^-1^). Heterotrophs and [cyanobacteri](app:ds:cyanobacteria)al cell densities were determined after incubation for 2 d. All the experiments were performed under aseptic conditions, the controls (CK) and the treatments were replicated three times, and the arithmetical means (± SD) were used as the ﬁnal results.

The sensitivities of the cyanobacterium and heterotrophs to antibiotics were shown in Table S1. It indicated that tetracycline, cephalosporin and streptomycin had obvious inhibiting effect on strain B905-1, while kanamycin and penicillin had no inhibiting effect. It was also demonstrated that *Microcystis* 905 was significantly inhibited by all of the tested antibiotics except penicillins. Although the associated heterotrophic bacteria could be inhibited by tetracycline, cephalosporin and streptomycin, the growth of *Microcystis* 905 was inhibited at the same time, hence, the antibiotics used in this study were unable to obtain axenic cyanobacterium.

Table S1 Effects of antibiotics on heterotrophs and [cyanobacteri](app:ds:cyanobacteria)um

| Antibiotics | Tetracycline | Cephalosporins | Kanamycin | Penicillins | Streptomycin |
| --- | --- | --- | --- | --- | --- |
| Halo of growth inhibition | 3.3 mm | 2.4 mm | — | — | 1.8 mm |
| Inhibition efficiency | 94.3 ± 3.5% | 88.5 ± 4.1% | 50.7 ± 2.4%* | 5.6 ± 0.2%** | 81.2 ± 3.9%** |

* and ** represent a statistically significant difference of *p* < 0.05 and *p* < 0.01 when compared to the control.

The results of lysozyme on [the cyanobacteri](app:ds:cyanobacteria)um and heterotrophic bacteria demonstrated that both *Microcystis* 905 and heterotrophic bacteria were reduced with the increasing of lysozyme concentration; moreover, the reduction of *Microcystis* 905was much more obvious (Fig. S1). As the lysozyme concentration increased from 0 to 10.0 mg mL^-1^, the cell number of [cyanobacteri](app:ds:cyanobacteria)um decreased much stronger than that of the heterotroph. Hence, these approaches were ineffective in completely separating the *Microcystis* from the heterotrophs.


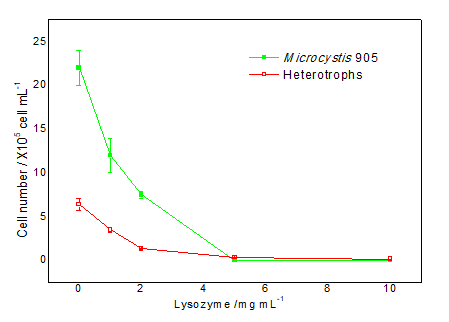


Fig. S1 Effects of lysozyme on heterotrophs and *Microcystis* 905
